# *In Vivo* Photoacoustic Imaging of Anterior Ocular Vasculature: A Random Sample Consensus Approach

**DOI:** 10.1038/s41598-017-04334-z

**Published:** 2017-06-28

**Authors:** Seungwan Jeon, Hyun Beom Song, Jaewoo Kim, Byung Joo Lee, Ravi Managuli, Jin Hyoung Kim, Jeong Hun Kim, Chulhong Kim

**Affiliations:** 1 Department of Creative IT Engineering, Pohang University of Science and Technology (POSTECH), 77 Cheongam-ro, Nam-gu, Pohang, Gyeongbuk 37673 Republic of Korea; 2 Department of Biomedical Sciences, Seoul National University College of Medicine, 103 Daehak-Ro, Jongno-Gu, Seoul, 03080 Republic of Korea; 3 Department of Bioengineering, University of Washington, Seattle, 98195 USA; 4 Hitachi Medical Systems of America, Twinsburg, OH 44087 USA; 5 Department of Ophthalmology, Seoul National University Hospital, 101 Daehak-Ro, Jongno-Gu, Seoul, 03080 Republic of Korea

## Abstract

Visualizing ocular vasculature is important in clinical ophthalmology because ocular circulation abnormalities are early signs of ocular diseases. Photoacoustic microscopy (PAM) images the ocular vasculature without using exogenous contrast agents, avoiding associated side effects. Moreover, 3D PAM images can be useful in understanding vessel-related eye disease. However, the complex structure of the multi-layered vessels still present challenges in evaluating ocular vasculature. In this study, we demonstrate a new method to evaluate blood circulation in the eye by combining *in vivo* PAM imaging and an ocular surface estimation method based on a machine learning algorithm: a random sample consensus algorithm. By using the developed estimation method, we were able to visualize the PA ocular vascular image intuitively and demonstrate layer-by-layer analysis of injured ocular vasculature. We believe that our method can provide more accurate evaluations of the eye circulation in ophthalmic applications.

## Introduction

Visualizing ocular vasculature is important in clinical ophthalmology because ischemia and neovascularization are early signs of various ocular diseases^1, 2^. Conventionally, slit-lamp biomicroscopy is used to evaluate such disorders, but it is not able to show vessels clearly when the vasculature is accompanied by opaque scar tissue. Fluorescence angiography is also commonly used for ocular vascular imaging, but the injection of the required contrast agents can inflict pain and create potential complications. Optical coherence tomography (OCT), another widely used tool nowadays, can provide high-resolution and volumetric images of both the ocular structure^3^ and the vasculature by encoding the intensity variance resulting from blood flow^4^.

Photoacoustic microscopy (PAM) is an emerging imaging technology that also enables vasculature visualization in 3D^5^ because of its innate depth-resolving imaging capability^6^. PAM can provide an ocular vascular image based on the inherent optical absorbance of hemoglobin itself^7^. Therefore, there is no side effect associated with exogenous contrast agent^8, 9^. Moreover, PAM. This unique strength of PAM can assist ophthalmic diagnosis by providing detailed morphologic information of the ocular vasculature^10^. As an example, consider corneal neovascularization, an excessive ingrowth of vessels into the cornea. Because the new corneal vessels can threaten the eyesight, it is important to assess the disease’s progression and treatment response accurately. In 2014, W. Liu *et al*.^11^ showed the segmentation of corneal neovascularization in an ocular PAM image by using the robust local regression smoothing. However, the classical smoothing techniques like least squares regression smooth out all datasets, so the fitting could be unstable and fail to detect corneal neovascularization when many outliers, generated from the new vessels, are included in the dataset^12^. In addition to corneal neovascularization, existing depth-visualizing methods have problems in observing the deep ocular vessels on curved surfaces covered with other blood vessels or hemorrhages because they show only sliced images at a certain depth^13^ or projected images^5^.

The existing visualization method shows only the projected image or the lateral slice image at a certain depth. Therefore, it is a problem to observe deep veins when the ocular blood vessels on the curved surface are obscured by other blood vessels or hemorrhages.

To overcome these difficulties, we propose a new technique for evaluating the anterior ocular vasculature, one that combines optical-resolution PAM (OR-PAM) and a surface estimation algorithm based on random sample consensus (RANSAC). RANSAC, a machine learning algorithm, is an alternative to classical fitting methods. This iterative parameter estimation can produce a very accurate model by computing only inliers, even if the number of outliers is significant^12^. Instead of roughly mapping the eye surface with the classical smoothing method, we use the RANSAC algorithm to find the eyeball’s center position and half-diameter. With the estimated ocular center position and half-diameter, by using the distance from the estimated eye surface we can reconstruct more intuitive depth-encoded images of the ocular vasculature than conventional depth-encoded images. From such a surface-based depth-encoded image, we successfully segmented the corneal new vessels induced after a chemical burn on a mouse eye and quantified the area of the neovascularization. Moreover, we confirmed that our new method could identify changes in deep vessels, even when the area was covered by limbal vessels and hemorrhages. Finally, histological validation agreed well with the PA imaging results. With the further development of our algorithm, we believe that PAM could provide quantitative vessel information to aid the diagnosis and monitoring of ocular diseases such as corneal neovascularization or limbal vessel ischemia.

## Results

### *In vivo* photoacoustic imaging of anterior ocular vasculature

**Figure 1:**
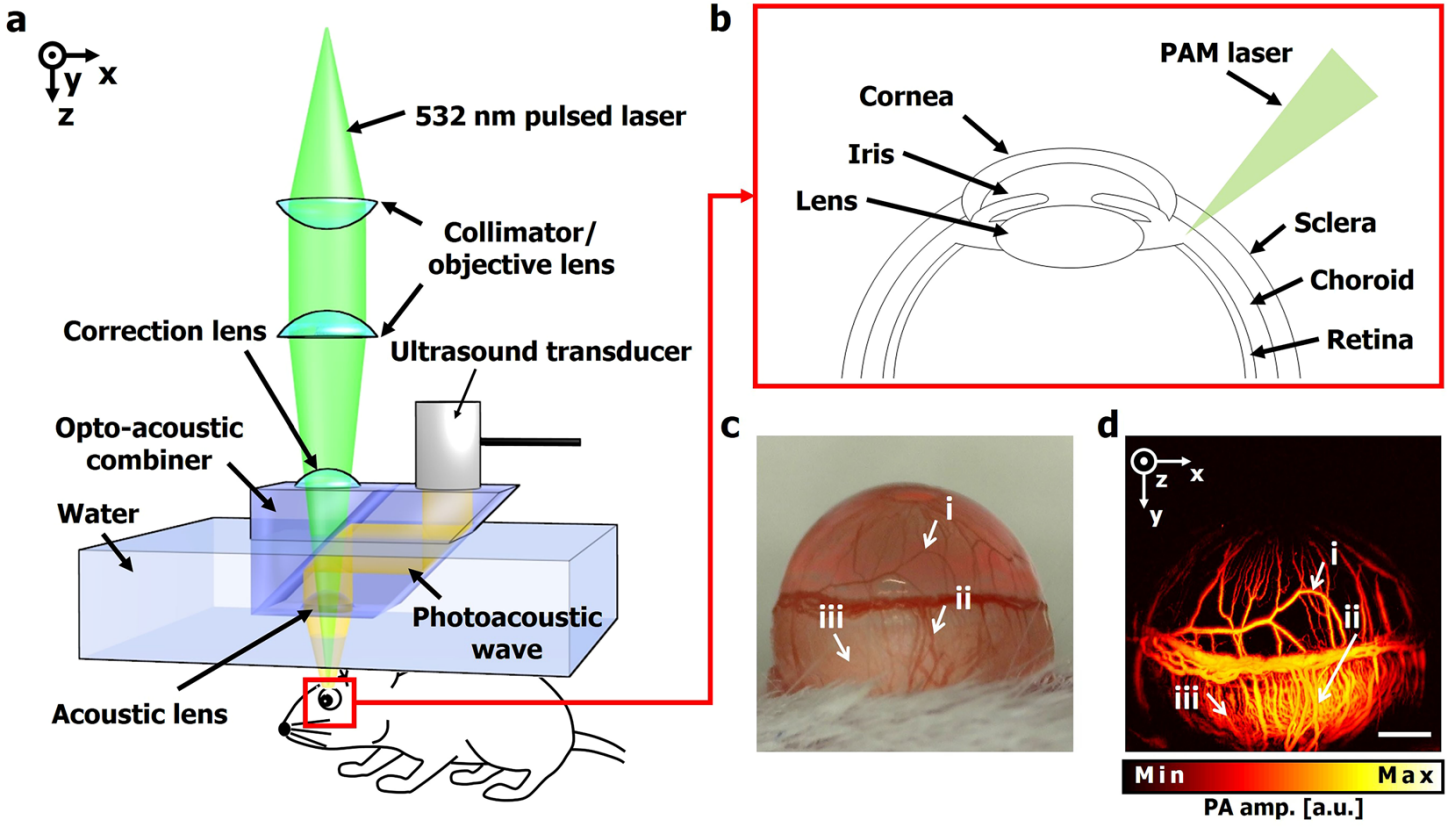
(**a**) Schematic of an optical-resolution photoacoustic microscopy (OR-PAM) system. (**b**) Diagram of a mouse eye. (**c**) Photograph (left) and PA MAP image (right) of a mouse eye. Scale bar: 500 μm. PA, photoacoustic; MAP, maximum amplitude projection.

We used a commercialized OR-PAM system (Switchable rapid-scanning PAM system, Microphotoacoustics, USA) (Fig. 1a) to image the mouse ocular vasculature. A detailed description of the system is provided in the Methods section. To monitor the change in ocular vasculature after chemical injury, a side view of the anterior segment was imaged, including the iris, limbus, and sclera, with a field-of-view of 3 mm × 3 mm (Fig. 1b). Then, the same region of the mouse eye was captured by a conventional digital microscope (AM4113ZTL, Dino-Light, Taiwan) (Fig. 1c). The OR-PAM maximum amplitude projection (MAP) image clearly shows the iris and limbal blood vessels as well as the choroidal and retinal vessels underlying the sclera (Fig. 1d). The vascular network matches well with the conventional microscopy image (highlighted with arrows (i) and (ii)). However, in the conventional microscopy image, the choroidal vessels are rarely visible due to the low contrast (highlighted with arrows (iii)).

### Eye surface estimation based on random sample consensus (RANSAC) and vessel visualization

**Figure 2:**
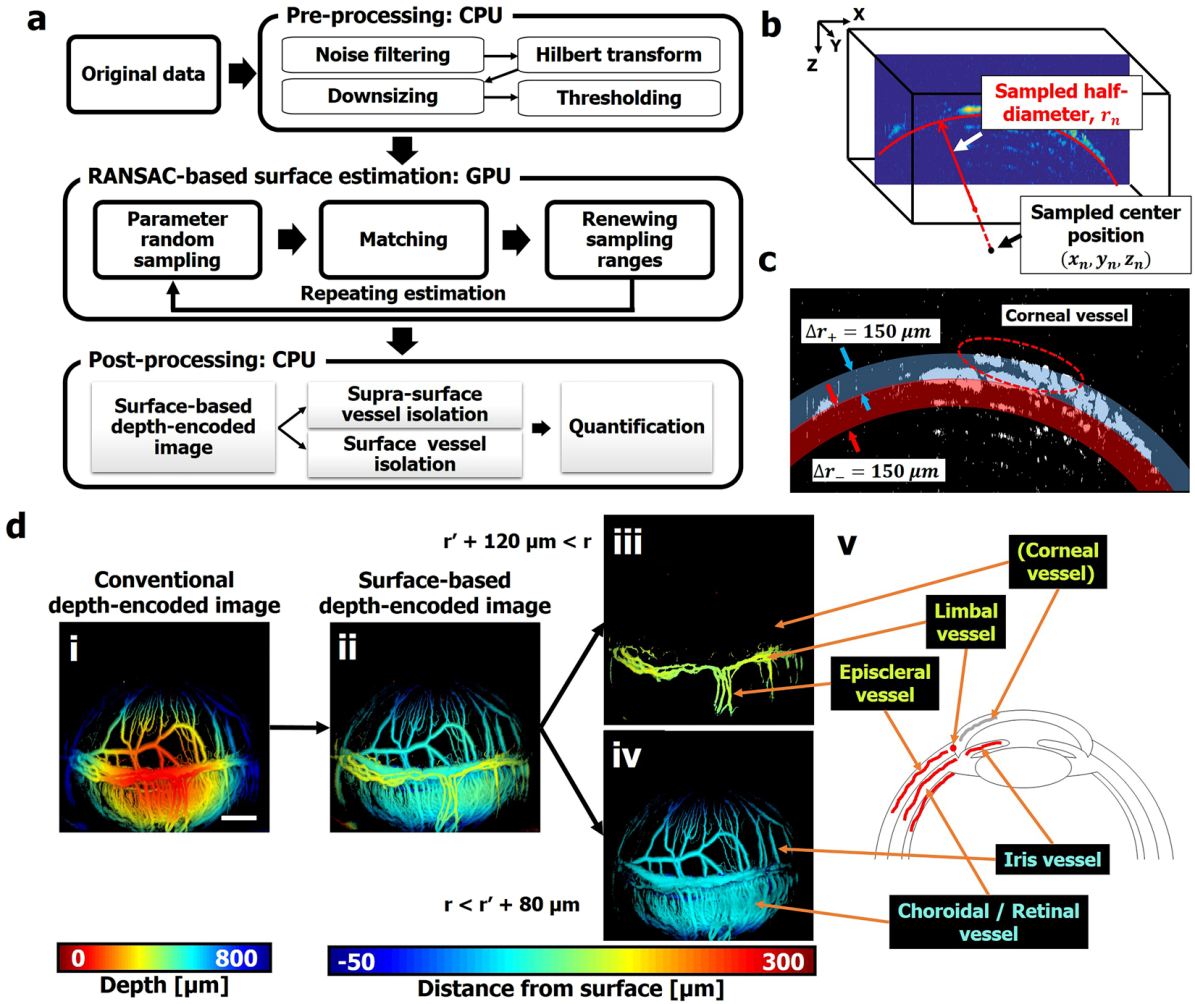
(**a**) Overall flowchart of RANSAC-based surface estimation algorithm. (**b**) RANSAC-based eye surface estimation concept. The red curved line is a surface of a randomly sampled sphere centered at $({x}_{n},{y}_{n},{z}_{n})$ with a half-diameter of ${r}_{n}$. (**c**) Pre-processed PA B-scan image of a mouse eye and a mask composed of positive and negative zones. (**d**) Conventional depth-encoded image (i) and surface-based depth-encoded image (ii) of a mouse anterior segment. Isolated vessels which are higher than r’+120 μm (iii) and lower than r’+80 μm (iv) from (ii), and corresponding vessels on a diagram (v). RANSAC, random sample consensus; PA, photoacoustic; r, distance from the estimated center position of a PA signal; and r’, estimated half-diameter of the eye. Scale bar: 500 μm.

We developed a RANSAC-based eye surface estimation method to reconstruct more intuitive ocular images than provided by conventional depth-encoded images and to analyze the ocular vasculature layer by layer. In using this method, to simplify the estimation we assumed that the mouse eye in the PA image had a perfect spherical structure centered at a specific position. Briefly, we randomly sampled 4000 sets of the eyeball center position and the half-diameter within specific random sampling ranges, then quantified how well the each set of parameters matched the acquired 3D PA data. After the 4000 matching processes, the random sampling ranges are reduced using the top 5 sets of parameters that best match the PA data. We repeated these processes to find the optimal center position and half-diameter. All of the above processes are divided into six steps: pre-processing, parameter random sampling, matching, renewing the random sampling ranges, repeating, and post-processing (Fig. 2a). Except for the preprocessing and post-processing steps, the surface estimation is accelerated by a graphics processing unit (GPU). We use a desktop computer with 64-bit Intel Core i7-4790 processor, 24 Gb RAM, and an NVIDIA GeForce GTX 970 video card. Detailed descriptions of each process step are given in the following:

*Step 1) Pre-processing the 3D PA image*: To increase the signal-to-noise ratio, the PA data is passed through a digital band-pass filter centered at the ultrasound center frequency and is enveloped by Hilbert transformation. The enveloped 3D data is downsized to reduce the calculation time. Then, we acquire the binary PA data, *P*(*x*, *y*, *z*), by thresholding the data to make the blood vessel signals into 1 s and the background into 0 s.

*Step 2) Parameter random sampling*: In this step, we randomly select 4000 parameter sets and each parameter set contains the eyeball’s center position and half-diameter. The number of the parameter sets is chosen to best use the constant memory size in the GPU. The initial random sampling ranges are selected as follows:

1 $$\begin{array}{c}{x}_{n}={c}_{x}+\xi [{d}_{x}]\,mm\\ {y}_{n}={c}_{y}+\xi [{d}_{y}]\,mm\\ {z}_{n}={c}_{z}+\xi [{d}_{z}]\,mm\\ {r}_{n}={c}_{r}+{\rm{\xi }}[{d}_{r}]\,mm,\end{array}$$

where the parameters $({x}_{n},{y}_{n},{z}_{n},{r}_{n})$ represent the center position along each axis (x, y, z) and the half-diameter of the n-th selected parameter set (Fig. 2b). Further, $({c}_{x},{c}_{y},{c}_{z},{c}_{r})$ are the centers of the random sampling ranges, $and\,\xi [{\rm{a}}]$ is a random real number that is uniformly distributed between –a and a. In this study, *c*_*x*_ and *c*_*y*_ are set to be a half of the PA image size because the eye is normally imaged at the center of the OR-PAM image. *c*_*z*_ is set to be 2.30 mm empirically based on the typical z-position of the eye, which depends on the focal depth of the OR-PAM system, $and\,{c}_{r}$ is set to be 1.70 mm according to the typical mouse eye size^14^. ${d}_{x},{d}_{y},{d}_{z},\,{\rm{and}}\,{d}_{r}$ are respectively set empirically to be 0.50, 0.50, 0.50, and 0.25. These initial random sampling ranges are consistently applied to all images.

*Step 3) Matching*: To find the parameter set nearest to the eyeball’s actual center position and half-diameter, all the parameter sets are matched with the preprocessed PA data. To estimate a surface including the innermost boundary of the vasculature, the PA data are matched to a mask, ${M}_{n}(x,y,z)$, composed of positive and negative zones defined as follows:

2 $${M}_{n}(x,y,z)=\{\begin{array}{c}1,if\,{r}_{n} < r < {r}_{n}+{\rm{\Delta }}{r}_{+}\,(positive\,zone)\\ -1,if\,{r}_{n}-{\rm{\Delta }}{r}_{-} < r < {r}_{n}(negative\,zone)\\ 0,else\,\end{array},$$

where *r* is the distance from the center position, $({x}_{n},{y}_{n},{z}_{n}$), and ${\rm{\Delta }}{r}_{+}$ and ${\rm{\Delta }}{r}_{-}$ are the thicknesses of the positive and negative zones, respectively (Fig. 2c). In this study, we set ${\rm{\Delta }}{r}_{+}$ and ${\rm{\Delta }}{r}_{-}$ to be 150 μm in order to include an arbitrary vessel in the PA data. Then we calculate the matching score,$\,{S}_{n}$, as follows:

3 $${S}_{n}=\sum _{x}\sum _{y}\sum _{z}(P(x,y,z)\times {M}_{n}(x,y,z))\cdot $$

After calculating the 4000 matching scores, we extract the parameter sets, $(x{\text{'}}_{m},y{\text{'}}_{m},z{\text{'}}_{m},r{\text{'}}_{m})$, having five highest matching scores among the 4000 parameter sets. *m* indicates the order of highest matching score varying from 1 to 5.

*Step 4) Renewing the random sampling ranges*:The random sampling ranges in Equation (1) are renewed by using the extracted top 5 parameter sets as follows:

4 $$\begin{array}{c}{x}_{n}=\langle x{\text{'}}_{m}\rangle +\xi [{\rm{\gamma }}\times std(x{\text{'}}_{m})+b]\,\,mm\\ {y}_{n}=\langle y{\text{'}}_{m}\rangle +\xi [{\rm{\gamma }}\times std(y{\text{'}}_{m})+b]\,\,mm\\ {z}_{n}=\langle z{\text{'}}_{m}\rangle +{\rm{\xi }}[{\rm{\gamma }}\times std(z{\text{'}}_{m})+b]\,\,mm\\ {r}_{n}=\langle r{\text{'}}_{m}\rangle +{\rm{\xi }}[{\rm{\gamma }}\times std(r{\text{'}}_{m})+b]\,\,mm,\end{array}$$

where $\langle {a}_{m}\rangle$ and $std({a}_{m})$ are respectively the average and standard deviation of ${a}_{m}$, γ is an adjustable scaling factor, and *b* is a constant variable to fix the minimum random sampling ranges. In this study, γ is set to be 2 and *b* is set to be 0.025 empirically.

*Step 5) Repeating*: With the renewed random sampling ranges, the processes from Step 2 to Step 5 are repeated until the nearest parameter set, $(x{\text{'}}_{1},y{\text{'}}_{1},z{\text{'}}_{1},r{\text{'}}_{1})$, is maintained for three repeats. We define that parameter set as the eye’s center position, $(x\text{'},y\text{'},z\text{'})$, and half-diameter, $r\text{'}$.

We next try the surface estimation from Step 2 to Step 5 ten times with a single PA dataset to find the average processing time and to evaluate the precision of our estimation algorithm. On average, for a single process of Step 3, it takes about 23,700 ms and 257 ms of CPU and GPU processing, respectively, and requires 9.5 repeats (38,000 parameter sets) to finish the estimation. Considering the additional time to initialize and finish the GPU processing, the total average processing time is 2.82 s (Table 1). Table 2 shows the average and standard deviation of the optimal parameters after the ten trials. We observe that the standard deviations of the all estimated parameters are less than 5 μm. In our estimation method, we used single precision, which has seven significant digits. Hence, the final precision is fixed to be 0.001 μm because the maximum range of the parameters is 3000 μm. However, as the minimum voxel size in our PA image was 2.5 μm, we rounded the resulting values in Table 2.

**Table 1:** Averaged GPU processing time to estimate surface.

Averaged processing time	
Memory allocation and copy	374 ms
Matching	257 ms
Required number of repeats	9.50
Memory deallocation	5.02 ms
**Total processing time**	2,820 ms

**Table 2:** Representative average and standard deviation of the optimal parameters.

Estimated parameter set	
$x{\text{'}}_{1}$	1275 ± 1 μm
$y{\text{'}}_{1}$	1183 ± 2 μm
$z{\text{'}}_{1}$	2477 ± 3 μm
$r{\text{'}}_{1}$	1748 ± 3 μm

(Average ± standard deviation, n = 10).

*Step 6) Post-processing*: A. Surface-based depth-encoded image.

We reconstruct the surface-based depth-encoded image, ${D}_{s}(x,y)$, by using the extracted parameter set, $(x\text{'},y\text{'},z\text{'},r\text{'})$, as follows:

5 $${D}_{s}(x,y)=\sqrt{{(x-x\text{'})}^{2}+{(y-y\text{'})}^{2}+{({D}_{c}(x,y)-z\text{'})}^{2}\,}-r\text{'},$$

where the conventional depth-encoded image, ${D}_{c}(x,y)$, represents the depth of the maximum PA signal in enveloped PA data,$\,{P}_{hilbert}(x,y,z)$, at (*x, y*) based on a scanning plane (Fig. 2d(i)) and is defined as follows:

6 $${D}_{c}(x,y)=argmax({P}_{hilbert}(x,y,z)).$$

Note that we thresholded the background of all conventional depth-encoded and surface-based depth-encoded images using the PA intensity. In contrast to the conventional depth-encoded image, the surface-based depth-encoded image, ${D}_{s}(x,y)$, represents the distance between the corresponding PA signal and the estimated eyeball surface in the direction normal to the surface (Fig. 2d(ii)). We visualize ${D}_{s}(x,y)$ within the range from −50 μm to −50 μm, saturating the signals whose distance is out of the range.

B. Supra-surface vessel isolation.

Normally, limbal vessels are located above the choroidal and retinal vessels, and corneal new vessels, induced by an injury on the anterior segment, grow along the outer eye surface. We segment the supra-surface vessels by thresholding the surface-based depth-encoded image. Figure 2d(iii) shows the segmented image with a threshold range of $r\text{'}+120\,\mu m < r$. We observe that only limbal vessels and episcleral vessels are detected in the supra-surface image. In addition, the corneal new vessels are also successfully segmented from the alkali-burned mouse eyes and described in the Results, Section 3.

C. Surface vessel isolation.

In a similar way to supra-surface vessel isolation, we segment the choroidal/retinal vessels and iris vessels that are located in a relatively lower layer than the vessels above the surface. We do this by projecting the PA signals within a certain range, $r\text{'} < r < r\text{'}+80\,\mu m$ (Fig. 2d(iv)). We also demonstrate on-the-surface vessel isolation within various ranges (see Supplementary Video S1). In this projection process, the PA signals below the estimated surface, $r\text{'}$, are excluded to eliminate reverberation artifacts.

### Supra-surface vessel isolation enables temporal and quantitative evaluation of corneal neovascularization

**Figure 3:**
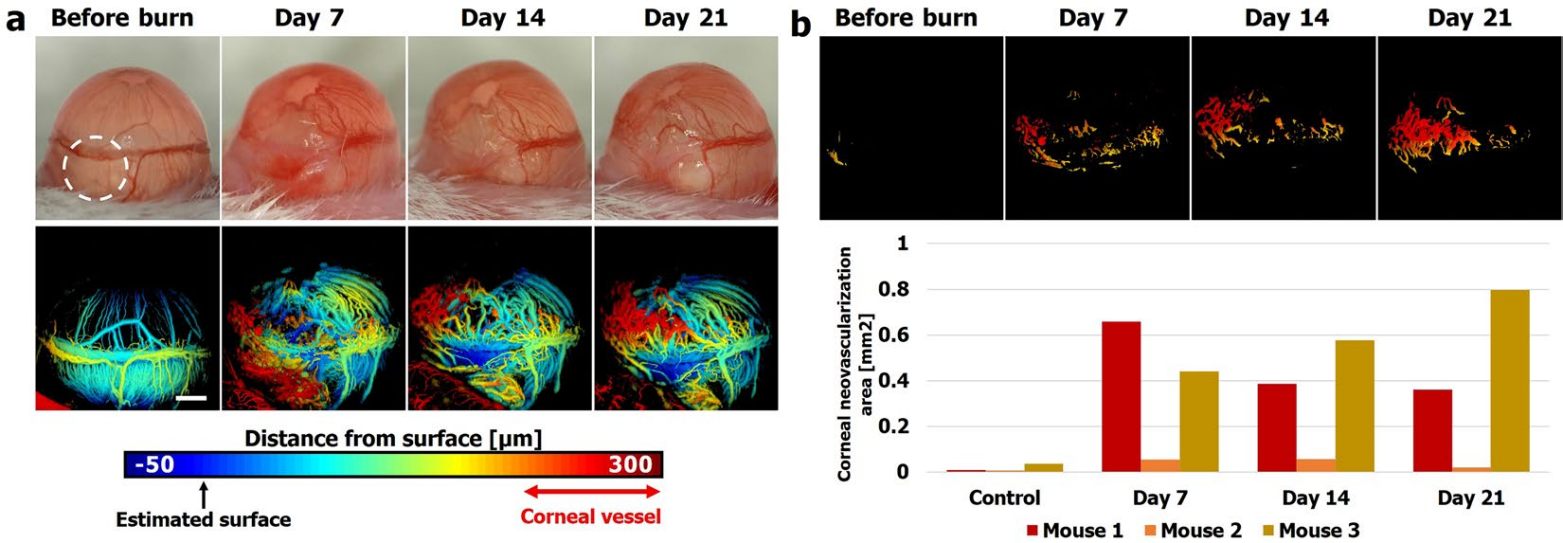
(**a**) Representative photographic images (upper row) and surface-based depth-encoded images (lower row) taken before alkali burn and 7, 14, and 21 days after alkali burn. The burned areas are highlighted with white dashed circles. (**b**) Images after supra-surface vessel isolation from the surface-based depth-encoded images (upper panel) and quantified corneal neovascularization area of the three mice after alkali burn (lower panel). Scale bar: 500 μm.

We have demonstrated isolation of vessels according to the surface estimated by the RANSAC-based algorithm. This processing is useful when vessels are stacked at the same position on the image plane. As an example, consider an alkali burn. Such a burn is known to induce corneal neovascularization^15^ on a different layer than that of the iris vessels. We inflicted an alkali burn to the eyes of mice, followed by evaluation with photographs and PAM images. The photograph on day 7 shows notable edema of the cornea and conjunctiva where the burn was given, and subsequent photographs show relief of the edema, leaving opacity behind (Fig. 3a, upper row). Interrupted visualization of the limbal vessels in the photographs could either reflect vessels damaged by the chemical burn or reflect undamaged vessels merely blocked from visualization by the overlying edema and opacity. In addition, neovascularization is detected throughout in the follow-up PAM images, but it is difficult to specify the layer and quantify the neovascularization from the photographs. In contrast, PAM could visualize vessels even in the area of edema and opacity. The surface-based depth-encoded images enabled tracking of vessels on the same layer, and revealed that limbal vessels are interrupted in the area of the chemical burn (Fig. 3a, lower row). Furthermore, neovascularization is shown above the estimated surface, and new vessels are growing upward along the same layer (Fig. 3a, lower row), which corresponds anatomically to the cornea. The surface-based depth-encoded images were further processed based on the distance from the estimated surface ($r\text{'}+180\,\mu m < r$), and the upper hemisphere, demarcated by the limbal vessel, was manually selected to isolate corneal vessels. The isolation of supra-surface vessels enables temporal follow-up of corneal neovascularization at a glance and clearly describes its progression, which starts from the limbus and grows toward the corneal center (Fig. 3b, upper row). Considering the distortion induced by projecting the curved surface to the image plane, we could calculate the actual area of cornel neovascularization and record its temporal progression (Fig. 3b, lower row). A detailed description of calculating the area from the images is given in the Methods section. We also implemented the isolation of the corneal neovascularization and quantified the area after an acid burn (see Supplementary Fig. S1). Two of the three corneal neovascularization areas increased by more than 0.5 mm^2^ after the alkali burn, but in all the mouse eyes there was no significant change of the detected area after the acid burn.

### Surface vessel isolation enables *in vivo* evaluation of choroidal and retinal vessels

**Figure 4:**
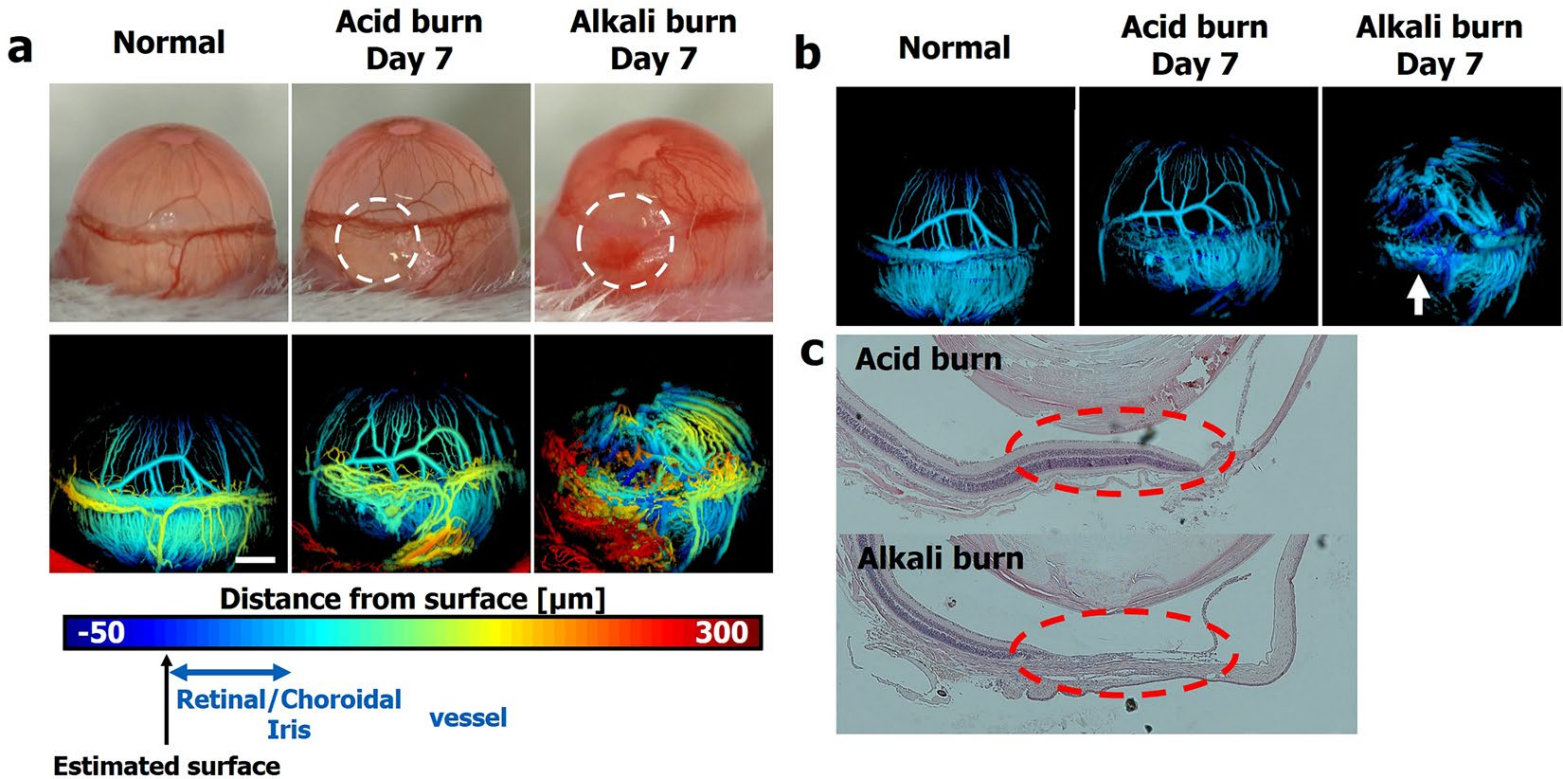
(**a**) Representative photograph images (upper row) and surface-based depth-encoded images (lower row) taken from the untreated eye, and eyes seven days after acid burn and alkali burn. The burned areas are highlighted with white dashed circles. (**b**) Images after surface vessel isolation from the surface-based depth-encoded images. (**c**) Histological sections from eyes after acid burn (upper panel) and alkali burn (lower panel). The burned areas are highlighted with red dashed circles. Scale bar: 500 μm.

Because photoacoustic imaging techniques enable imaging deep structures^16, 17^, OR-PAM can visualize choroidal and retinal vessels beneath the sclera that forms the opaque outermost layer of an eyeball and prevents visualization of vessels underneath it. To evaluate the capability of our PAM system to assess deep vessels, we prepared normal eyes, eyes after an alkali burn, and eyes after an acid burn. Surface-based depth-encoded PAM images of the normal eye could visualize intact choroidal/retinal vessels that could not be clearly seen in the photographs (Fig. 4a). Conjunctival swelling in both eyes after chemical burns (to a lesser extent in eyes after acid burn) and hemorrhages in eyes after alkali burns made visualization of deep vessels even more difficult (Fig. 4a, upper row). Surface-based depth-encoded PAM images can to some degree visualize choroidal and retinal vessels, but it is still difficult to evaluate them due to overlying limbal vessels and hemorrhages (Fig. 4a, lower row). Thus, we isolated deep vessels by applying a threshold to the surface-based depth-encoded PAM images ($r\text{'} < r < r\text{'}+65\,\mu m$). The results clearly show the iris and choroidal/retinal vessels and provide evidence of damage to deep vessels (Fig. 4b). They even provide information about the damaged area (arrow) without the necessity of tissue preparation or injection of contrast labels. To confirm the depth of damages, histologic assessments were performed. The acid-burned eyes show intact layers of the retina and choroid, while the alkali-burned eyes show structural disintegration that extends deep into the full thickness of the retina (Fig. 4c). These results well agree with the previously report results that acids are generally less harmful than alkalis because coagulated proteins prevent acid penetration^18^.

## Discussion and Conclusions

We have developed a RANSAC-based eye surface estimation method, designed to find the eyeball’s center position and half-diameter, to analyze ocular vasculature layer by layer. The RANSAC algorithm is a very powerful parameter estimation technique using an iterative random sampling process. However, due to the nature of random-based sampling, RANSAC cannot always find the optimal set from the input dataset. In other words, the center position and half-diameter of the eye estimated via RANSAC could change whenever we estimate the parameters again. This serious problem might lead to inconsistent and unreliable results in both visualization and segmentation. Increasing the number of iterations is needed to minimize the probability of the problem. For this reason, we designed this estimation method to repeat the 4000 matching processes until the nearest parameter set is maintained for three repeats, and we obtain a precise estimation result with a standard deviation of less than 5 μm for all parameters (i.e., *x, y, z*, and *r*; Table 2). As mentioned, the high precision of RANSAC-based estimation requires a large number of iterations and a long computation time, especially when 3D data is addressed, as in this study. However, we have demonstrated GPU-based acceleration of this computation, improving the processing speed by approximately 90 times (to within 3 seconds on average) over CPU processing (Table 1). Although the GPU processing speeds up the processing, it would be difficult to evaluate the ocular vasculature immediately because of the slow speed of the scanning system used in this experiment (about 1 Hz/B-scan). However, we believe that this problem could be solved by using a fast scanning OR-PAM system (~250 Hz/B-scan)^19–21^.

Using the eyeball’s estimated center position and half-diameter, we have reconstructed a surface-based depth-encoded ocular PA image and compared it with a conventional depth-encoded image. The conventional depth-encoded image represents the signal depth from a scanning plane. This image might be helpful in understanding the 3D structure of flat samples, such as a mouse ear or cells^22, 23^ because the surface of the sample and the scanning plane are approximately parallel, but it is not easy to understand the structure when the sample surface is curved, like the ocular vasculature (Fig. 2d(i)). Taking a different approach, the surface-based depth-encoded image represents the signal depth from the estimated ocular surface, so it is easier to understand the ocular vasculature. In Fig. 2d(ii), we observe that the choroidal/retinal vessels and iris vessels are rendered in the same blue, whereas the limbal vessels are rendered in yellow. The color difference makes it clear that the limbal vessels are located on a higher layer than the other vessels. Anatomically, the actual shape of an ocular anterior segment, including the iris vessels and the choroidal/retinal vessels, is not a perfect sphere but close to an ellipse, and human eyes have a longer elliptical shape than mouse eyes^14, 24^. Hence, an estimation error could occur when applying our algorithm to human eye images. However, we expect that this problem can be solved by considering several parameters, such as the radius of the iris and the anterior choroid curvature, separately in the estimation process. Additional time for processing would be required.

We have demonstrated temporal and quantitative analysis of corneal neovascularization. Because corneal neovascularization is present in most cases of blindness related to corneal disease^25^, it is important to assess its progression and treatment response. Conventionally, corneal neovascularization is evaluated with slit-lamp biomicroscopy or photographs taken during slit-lamp examinations^26, 27^. However, the visibility largely depends on the quality of images, and even in good quality images some vessels are not visible when accompanied by opaque scar tissue^28^. Recently, fluorescein and indocyanine green angiography have been utilized in some studies^29^, but their invasiveness and the risks of serious adverse reactions have hindered their wide use^30^. Our system provides high contrast images without labeling and additionally provides automated, objective quantification of corneal neovascularization within a few seconds after image acquisition. With the help of the RANSAC-based algorithm, we can evaluate choroidal and retinal vessels that lie deep from the surface. Photoacoustic imaging is valuable in imaging deep structures, but analysis of reconstructed 3D volume images and conventionally depth-encoded images is not intuitive. The RANSAC-based algorithm enables layer by layer analysis of multi-layered structures with curvature, and the resultant images can demonstrate temporal changes to users intuitively, as shown in Fig. 4b. The peripheral choroid has not received much attention from ophthalmologists, despite the presence of a watershed zone in the far temporal sector of the human choroid^31^. The watershed zone suggests that damages such as an alkali burn inflicted on the peripheral retrograde choroidal arteries can lead to choroidal ischemia in the area from the ora serrata to the watershed zone. Despite its significance, assessment of the peripheral choroid from inside is time-consuming and troublesome: Visualizing the choroidal vessels requires a 30-diopter lens to expand the angle of view and indocyanine green for angiography^31^. In comparison, by imaging from the outside, our approach facilitates peripheral choroid evaluation.

We conducted *in vivo* photoacoustic imaging of anterior ocular vasculatures with the help of RANSAC-based ocular surface estimation algorithm. The algorithm proved very useful for evaluating multi-layered and curved structures such as eyes. We have demonstrated intuitively understandable layer-by-layer images reconstructed from 3D volume photoacoustic data of the anterior ocular vasculature. The resultant isolation of supra-surface vessels and surface vessels enables *in vivo* temporal and quantitative evaluation of corneal neovascularization and temporal evaluation of choroidal/retinal vessels, respectively, in the mouse model of chemical burn. We believe that our approaches will help expand the applications of PAT in ophthalmology. The future direction of this study is to apply this technology to human eyes. However, besides the problem of the opaque sclera and the anatomical differences of the eyeballs, there are some additional problems that the imaging speed is too slow and the coupling medium should be used. Recently, a non-contact OR-PAM system with a high scan rate and an improved signal-to-noise (SNR) has been developed. We believe that by using the non-contact imaging method, the problems could be solved^32^.

## Methods

### Optical-resolution photoacoustic microscopy (OR-PAM) system

To image the mouse eyes, we utilized a commercial OR-PAM (Switchable rapid-scanning PAM system, Microphotoacoustics, USA). For photoacoustic irradiation, we used a Nd:YAG 532 nm laser (AWAVE 532-1W-10K, Advanced Optowave, USA; 10 ns laser pulse width). The objective numerical aperture (NA) was 0.1, so the calculated lateral resolution of the system was about 3 μm. The generated photoacoustic waves were detected by an ultrasonic transducer (V214-BB-RM, Olympus-NDT, USA; 50 MHz center frequency; 50 MHz bandwidth:) and acquired by a data acquisition board (ATS9350, AlazarTech, Canada) with a sampling rate of 500 MHz.

We applied the American National Standard Institute safety standards (ANSI Z136.1-2000) to calculate the maximum permissible exposure (MPE) for ocular exposures^8^. The MPE for a single laser pulse was MPE_SP_ = 5.0 *C*_*E*_ × 10^−7^ = 1.33 × 10^−4^ J/cm^2^, where *C*_*E*_ is a correction factor calculated as 267 according to the NA (0.1) of our system. The repetitive pulse limit was then calculated as MPE_RP_ = n_total_^−0.25^ ∙ MPE_SP_ = (7.2 × 10^5^)^−0.25^ ∙ 1.33 × 10^−4^ J/cm^2^ = 4.6 × 10^−6^ J/cm^2^, where n_total_ was the total number of pulses during imaging (1200 × 600 pixels in the x and y direction, respectively). Therefore, the maximum permissible single laser pulse energy in a typical human pupil of 7 mm was calculated as MPE_RP_ × *pupil area* = 1.8 μJ, which was 15 times higher than the experimentally used pulse energy of 120 nJ. In addition, laser safety on the scleral surface was also considered^33^. We assumed the laser focus was approximately 500 μm because we optically focused on the choroidal vessel just below the sclera, typically 500 μm^34^. The diameter of the irradiated region was calculated as 106 μm by the Gaussian beam profile and the x-axis step size was 2.5 μm, so the repetitive pulse number on the irradiated region, n, was 42. Thus, the repetitive pulse limit at the scleral surface was MPE_RP_ = n^−0.25^ × MPE_SP_ = 42^−0.25^ × 20 mJ/cm^2^ = 7.9 mJ/cm^2^, where MPE_SP_ was a single pulse MPE at the skin. Therefore, the maximum permissible pulse energy was calculated to be about 700 nJ, which is about 6 times larger than the energy we used.

### Animal preparation

All animal experimental procedures were conducted following the laboratory animal protocol approved by the institutional animal care and use committee of the Pohang University of Science and Technology (POSTECH). All animal experiments were performed in accordance with the National Institutes of Health Guide for the Care and Use of Experimental Animals. We used three pairs of female BALB/c mice in this experiment. The mice were initially anesthetized with 4% isoflurane vaporized by inhalation gas (1.0 L/min flow rate) and kept under anesthesia with 1% isoflurane during alkali-/acid-burning and imaging. Then, the mouse was placed on an animal stage with a silicon-heating pad to maintain the body temperature of the mice during imaging. We used ultrasound gel on the mouse eye as a coupling medium between the eye and a water tank. Alkali- and acid-burn injuries were performed on the right eyes. To regulate the injury area, we used 1 mm diameter disks of Whatman paper which were soaked with 1 N NaOH solution for alkali-burn and 1 N HCl solution for acid-burn. The soaked pieces of paper were placed on the eye for 30 seconds, then the corneal surface was rinsed with 20 mL of physiological saline solution. We imaged the eyes on days 0 (control and after burn), 7, 14, and 21 after chemical burn to observe the healing process. To observe the reactions near the limbal blood vessels after the alkaline and acid burns, we imaged near the limbus, including limbal, iris and choroidal blood vessels. During PA imaging, we pressed the area around the eye slightly to make the area more visible.

### Quantification of corneal neovascularization

To quantify the corneal neovascularization, we calculated the new vessel area considering the slope of the eye surface (see Supplementary Fig. S2). Because the detected new vessels were usually located on the spherical eye surface, the actual area of the corneal vessels became distorted when they projected in a 2D image. To correct the projected area, we calculated a cosine feature map, $C(x,y)$, representing the angle of the surficial slope at $(x,y)$. We assumed that the actual surface on the sphere, corresponding to each projected pixel, was flat to simplify the calculation:

7 $$C(x,y)=\frac{(z{\text{'}}_{1}-{D}_{t}(x,y))}{(r{\text{'}}_{1}+{D}_{s}(x,y))}.$$

Finally, the corrected corneal new vessel area, ${A}_{CNV}$, was calculated by

8 $${A}_{CNV}={A}_{pixel}\sum _{x}\sum _{y}{B}_{CNV}(x,y)/C(x,y),$$

where ${A}_{pixel}$ is the area of a projected pixel and ${B}_{CNV}(x,y)$ is the binary image of segmented corneal new vessels within the threshold range $r < r^{\prime} +180\,\mu m$.

## Electronic supplementary material

Video S1

Supplementary Information

## References

[1] Spaide RF, Armstrong D, Browne R (2003). Choroidal neovascularization in age-related macular degeneration–what is the cause?. Retina.

[2] Flammer J (2002). The impact of ocular blood flow in glaucoma. Progress in retinal and eye research.

[3] Lee C (2014). Stimulated penetrating keratoplasty using real-time virtual intraoperative surgical optical coherence tomography. Journal of biomedical optics.

[4] de Carlo TE, Romano A, Waheed NK, Duker JS (2015). A review of optical coherence tomography angiography (OCTA). International Journal of Retina and Vitreous.

[5] Jeon M, Kim J, Kim C (2016). Multiplane spectroscopic whole-body photoacoustic imaging of small animals *in vivo*. Medical & biological engineering & computing.

[6] Zhang Y (2014). Non-invasive multimodal functional imaging of the intestine with frozen micellar naphthalocyanines. Nature nanotechnology.

[7] Deán-Ben, X. L., Bay, E. & Razansky, D. Functional optoacoustic imaging of moving objects using microsecond-delay acquisition of multispectral three-dimensional tomographic data. *Scientific reports* **4** (2014). 10.1038/srep05878 PMC4115207 25073504

[8] Hu S, Rao B, Maslov K, Wang LV (2010). Label-free photoacoustic ophthalmic angiography. Optics letters.

[9] de La Zerda A (2010). Photoacoustic ocular imaging. Optics letters.

[10] Jeon M, Kim C (2013). Multimodal photoacoustic tomography. IEEE transactions on multimedia.

[11] Liu W (2014). *In vivo* corneal neovascularization imaging by optical-resolution photoacoustic microscopy. Photoacoustics.

[12] Fischler MA, Bolles RC (1981). Random sample consensus: a paradigm for model fitting with applications to image analysis and automated cartography. Communications of the ACM.

[13] Zhang E, Laufer J, Pedley R, Beard P (2009). *In vivo* high-resolution 3D photoacoustic imaging of superficial vascular anatomy. Physics in medicine and biology.

[14] Remtulla S, Hallett P (1985). A schematic eye for the mouse, and comparisons with the rat. Vision research.

[15] Paranthan RR, Bargagna-Mohan P, Lau DL, Mohan R (2011). A robust model for simultaneously inducing corneal neovascularization and retinal gliosis in the mouse eye. Molecular Vision.

[16] Pramanik M, Kim C (2015). Looking Deeper: Multimodal and contrast-enhanced photoacoustic imaging offer a clearer view within tissues for more accurate diagnosis. IEEE pulse.

[17] Kim, J. *et al*. Programmable Real-time Clinical Photoacoustic and Ultrasound Imaging System. *Scientific Reports* **6** (2016). 10.1038/srep35137 PMC5059665 27731357

[18] Mead, M. D. & Colby, K. A. Evaluation and initial management of patients with ocular and adnexal trauma in *Principles and Practice of Ophthalmology* (ed. Daniel M. Albert) 3361–3382 (WB Saunders Philadelphia, 1994).

[19] Kim JY, Lee C, Park K, Lim G, Kim C (2015). A PDMS-based 2-axis waterproof scanner for photoacoustic microscopy. Sensors.

[20] Kim, J. Y., Lee, C., Park, K., Han, S. & Kim, C. High-speed and high-SNR photoacoustic microscopy based on a galvanometer mirror in non-conducting liquid. *Scientific Reports* **6** (2016). 10.1038/srep34803 PMC5052531 27708379

[21] Kim, J. Y., Lee, C., Park, K., Lim, G. & Kim, C. Fast optical-resolution photoacoustic microscopy using a 2-axis water-proofing MEMS scanner. *Scientific reports* **5** (2015). 10.1038/srep07932 PMC4300456 25604654

[22] Yao J (2012). Double-illumination photoacoustic microscopy. Optics letters.

[23] Yao J (2016). Multiscale photoacoustic tomography using reversibly switchable bacterial phytochrome as a near-infrared photochromic probe. Nature methods.

[24] Panda-Jonas S, Jonas JB, Jakobczyk M, Schneider U (1994). Retinal photoreceptor count, retinal surface area, and optic disc size in normal human eyes. Ophthalmology.

[25] Lee P, Wang CC, Adamis AP (1998). Ocular neovascularization: an epidemiologic review. Survey of ophthalmology.

[26] Cheng, S.-F. *et al*. Short-term topical bevacizumab in the treatment of stable corneal neovascularization. *American journal of ophthalmology* **154**, 940–948. e941 (2012). 10.1016/j.ajo.2012.06.007 PMC3498533 22967868

[27] Trikha, S., Parikh, S., Osmond, C., Anderson, D. & Hossain, P. Long-term outcomes of Fine Needle Diathermy for established corneal neovascularisation. *British Journal of Ophthalmology*, bjophthalmol–2013–303729 (2014). 10.1136/bjophthalmol-2013-303729 24457357

[28] Anijeet DR (2012). Imaging and evaluation of corneal vascularization using fluorescein and indocyanine green angiography. Investigative ophthalmology & visual science.

[29] Spiteri N (2015). Corneal angiography for guiding and evaluating fine-needle diathermy treatment of corneal neovascularization. Ophthalmology.

[30] Cai, Y., del Barrio, J. L. A., Wilkins, M. R. & Ang, M. Serial optical coherence tomography angiography for corneal vascularization. *Graefe’s Archive for Clinical and Experimental Ophthalmology*, 1–5 (2016). 10.1007/s00417-016-3505-9 27722920

[31] Takahashi K, Muraoka K, Kishi S, Shimizu K (1996). Watershed zone in the human peripheral choroid. Ophthalmology.

[32] Hajireza P, Shi W, Bell K, Paproski RJ, Zemp RJ (2017). Non-Interferometric Photoacoustic Remote Sensing Microscopy Running title: Photoacoustic Remote Sensing. Light: Science & Applications.

[33] Rao, B., Hu, S., Li, L., Maslov, K. & Wang, L. V. *In vivo* label-free photoacoustic microscopy of the anterior segment of the mouse eye. *BiOS*, 75643E-75643E-75645 (2010).

[34] Vurgese S, Panda-Jonas S, Jonas JB (2012). Scleral thickness in human eyes. PloS one.

