# Supplementary material for: In Vivo Photoacoustic Imaging of Anterior Ocular Vasculature: A Random Sample Consensus Approach
Source: Sci Rep. 2017 Jun 28;7:4318. doi: 10.1038/s41598-017-04334-z (PMC5489523; doi:10.1038/s41598-017-04334-z)
Supplement: Supplementary file 2 — Supplementary Information [file 41598_2017_4334_MOESM2_ESM.pdf]

Supplementary Information for

In Vivo Photoacoustic Imaging of Anterior Ocular Vasculatures A Random Sample Consensus Approach

Seungwan Jeon[1†], Hyun Beom Song[2†], Jaewoo Kim[1], Byung Joo Lee[2], Ravi Managuli[3,4], Jin Hyoung Kim[5], Jeong Hun Kim[2,5*], and Chulhong Kim[1*]

[1]Department of Creative IT Engineering, Pohang University of Science and Technology (POSTECH), 77 Cheongam-ro, Nam-gu, Pohang, Gyeongbuk, 37673, Republic of Korea

[2]Department of Biomedical Sciences, Seoul National University College of Medicine, 103 Daehak-Ro, Jongno-Gu, Seoul 03080, Republic of Korea

[3]Department of Bioengineering, University of Washington, Seattle, 98195 USA

[4]Hitachi Medical Systems of America, Twinsburg, OH 44087 USA

[5]Department of Ophthalmology, Seoul National University Hospital, 101 Daehak-Ro, Jongno-Gu, Seoul 03080, Republic of Korea

[†]These authors contributed equally to this work.

*Corresponding author: chulhong@postech.edu and steph25@snu.ac.kr

The supplementary information includes Figure S1, Figure S2. and Video S1.

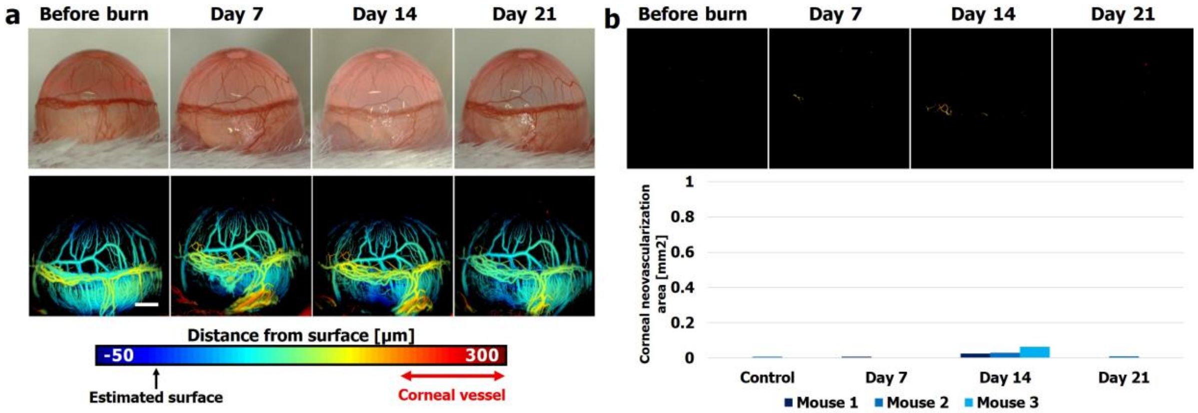

Figure S1. (a) Representative photographic images (upper row) and surface-based depth-encoded images (lower row) taken before acid burn and 7, 14, and 21 days after acid burn. (b) Images after supra-surface vessel isolation from the surface-based depth-encoded images. Scale bar: 500 μm

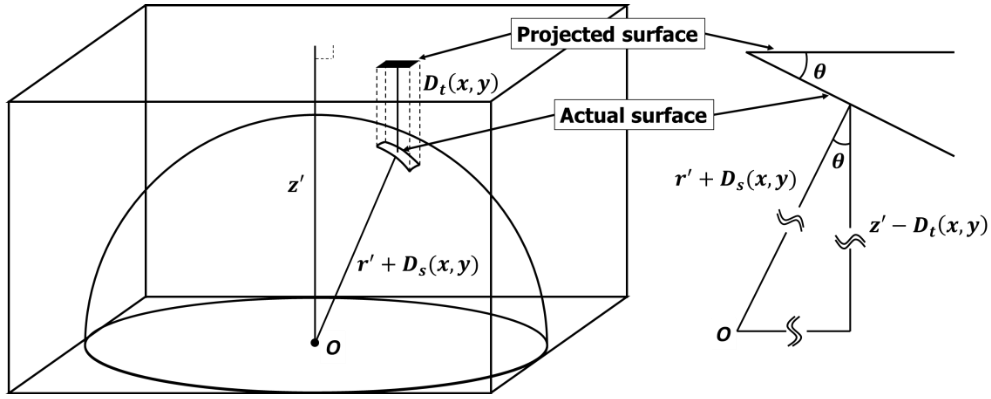

Figure S2. Correction of projected surface area.

Video S1. Surface vessel isolation with various depth thresholding ranges. Corresponding range is indicated at left-top side of the video.
